# Characterization and Analysis of the Carbonation Process of a Lime Mortar Obtained from Phosphogypsum Waste

**DOI:** 10.3390/ijerph18126664

**Published:** 2021-06-21

**Authors:** María Isabel Romero-Hermida, Antonio María Borrero-López, Vicente Flores-Alés, Francisco Javier Alejandre, José María Franco, Alberto Santos, Luis Esquivias

**Affiliations:** 1 Department Física de la Materia Condensada, Universidad de Sevilla, Avda. Reina Mercedes s/n, 41012 Sevilla, Spain; isaromerohermida@us.es (M.I.R.-H.); luisesquivias@us.es (L.E.); 2 Pro2TecS—Chemical Process and Product Technology Centre, Department of Chemical Engineering, ETSI, Campus de El Carmen s/n, University of Huelva, 21071 Huelva, Spain; am.borrero@diq.uhu.es (A.M.B.-L.); franco@uhu.es (J.M.F.); 3 Department Construcciones Arquitectónicas II, Universidad de Sevilla, Avda. Reina Mercedes n°4, 41012 Sevilla, Spain; falejan@us.es; 4 Department de Ciencias de la Tierra, Universidad de Cádiz, Campus Universitario de Pto, Real, Puerto Real, 11510 Cádiz, Spain; alberto.santos@uca.es

**Keywords:** lime putty, mortar, carbonation, rheology, ultrasound

## Abstract

This work addresses the reuse of waste products as a raw material for lime putties, which are one of the components of mortar. 1:3 Lime/sand mortars very similar to conventional construction mortars were prepared using a lime putty obtained from the treatment of phosphogypsum with sodium hydroxide. The physical, rheological and mechanical properties of this phosphogypsum-derived mortar have been studied, as well as the mineralogical composition, microstructure by scanning electron microscope (SEM) and curing process by monitoring carbonation and ultrasonic propagation velocity. Considering the negative influence of sulphates on the hardened material, the behaviour of the material after sulphates precipitation by adding barium sulphate was additionally tested. Carbonation progressed from the outside to the inside of the specimen through the porous system by Liesegang rings patterns for mortars with soluble sulphates, while the carbonation with precipitated sulphates was controlled by diffusion-precipitation. Overall, the negative influence of low-sulphate contents on the mechanical properties of mortars was verified. It must be highlighted the importance of their precipitation to obtain adequate performance.

## 1. Introduction

The industrial production of phosphoric acid is carried out from the treatment of calcium phosphate rocks with sulfuric acid. The chemical reaction of the industrial wet process occurs as follows [1]:

Ca_3_(PO_4_) + 3H_2_SO_4_ + 6H_2_O → 2H_3_PO_4_ + 3(CaSO_4_ · 2H_2_O) (1)

The majority of which is calcium sulphate dihydrate, i.e., gypsum (CaSO_4_·2H_2_O), called phosphogypsum (PG). The amount and chemical composition of the phosphogypsum obtained depend on the quality of the phosphate rock and the industrial process being chosen. Usually, there are about 3–7 tons of phosphogypsum per ton of wet-process phosphoric acid solution [2]. Currently, the storage of PG is more than 2000 Mt worldwide [3]. In the world, PG is produced at a rate of ~200 Mt/year of PG [4]. Its reuse for commercial applications is limited since the PG contains relatively high concentrations of toxic substances, such as fluorides and ammonium [5], heavy metals and radionuclides [4] coming from phosphate rock. Today, only 15% of all generated PG is recycled [1].

For the case we consider here, in which PG is treated with sodium hydroxide (Na(OH)) to obtain a lime putty of calcium hydroxide (Ca(OH)_2_) that reacts spontaneously with CO_2_ to form calcite (CaCO_3_) [5,6], the PG by-products could be considered in the same way as they were building wastes.

This process not only allows the reuse of PG, but also helps to mitigate the increasing concentration of CO_2_ in the atmosphere recycling a hazardous waste [7].

Calcium hydroxide is mainly used as a soil stabilizer but also to fabricate lime mortars. The degree of cohesion relies on its cementing capacity when carbonating by reaction with ambient CO_2_ [8]. The carbonation of Ca(OH)_2_ is a natural process and is affected by natural variables. It is caused by CO_2_ diffusion through the mortar porosity. The advance of carbonation front depends mainly on humidity, being more favorable when it is between 50–70%. Above or below this limit, conditions do not favour the carbonation of lime. Furthermore, carbonation is also influenced by the permeability of the material and the concentration of atmospheric CO_2_ [9].

One of the key characteristics of mortars is their workability, which is determined by their rheological properties [10,11], influencing at a great extent the performance of the final hardened product. In fact, the rheological properties of mortars are extremely important since several factors, related to mortar execution, consolidation, durability and strength, depend on the flow properties of the mortar paste.

In this work, the reuse of lime putty obtained from the treatment of PG and its possible applications are addressed. The study is carried out from the characterization of a mortar made with the lime putty resulting from the aforementioned PG treatment with caustic soda. This product should not negatively influence the rheological behavior of the fresh material and should provide a mechanical strength similar to that found in mortars prepared with traditional lime, not developing degradation reactions and ensuring adequate durability [12,13]. The evaluation of the behavior of this lime mortar, both in fresh and hardened state, by means of its rheological study and property testing, provides information on the response that this lime putty would have under conditions or applications like those of other commercial and/or traditional limes.

## 2. Materials and Methods

### 2.1. Starting Materials

Approximately, 6 kg of PG were collected from the PG stockpiles in Huelva (Spain) by courtesy of Fertiberia, S.A. This PG was oven-dried (40 °C) until completely dry and then milled and homogenized.

The major components and traces of the phosphogypsum sample, measured by X-ray fluorescence (XRF), are shown in Table 1. Besides, the activity concentrations of the natural radionuclides, which was measured by high-resolution gamma-ray spectrometry [14], were also included. The results indicated that PG is mainly composed of S (46.00 wt.% as SO_3_) and Ca (32.00 wt.% as CaO). The main impurities of the PG are Si (2.52 wt.% as SiO_2_), P (0.65 wt.% as P_2_O_5_), and Al (0.20 wt.% as Al_2_O_3_). This waste has been deeply analyzed and its characteristics and properties can be consulted elsewhere [8,11].

### 2.2. Synthesis of the Lime Putty

The procedure starts with the dissolution of raw PG in a sodium hydroxide (Na(OH)) solution that results in the precipitation of a whitish solid phase and a supernatant liquid. The precipitate corresponds mostly to portlandite (calcium hydroxide, Ca(OH)_2_), and subsequent evaporation of the supernatant liquid indicates that it mostly contains thenardite (sodium sulphate, Na_2_SO_4_), as expected from the following reaction:

CaSO_4_ · 2H_2_O + 2Na(OH) → Ca(OH)_2_ + Na_2_SO_4_ + 2H_2_O (2)

Full details of the methodology can be found elsewhere [11].

Since the remaining thenardite (a soluble sulphate) in the lime would generate efflorescence [15] in the resulting mortars, the barium hydroxide method was applied to convert it into barite (BaSO_4_) [13], whose solubility coefficient is log K_sp_ = −9.96 at 25 °C, vs. 2.67 of the thenardite.

Three hundred g of the lime putty obtained in the first stage are taken and then an alkaline solution of barium hydroxide (Ba(OH)_2_ · 8H_2_O, supplied by Panreac, Barcelona, Spain) was added in a molar ratio. [Ba^2+^]/[SO_4_^2−^] = 1.4 in 700 mL of distilled water. The reaction associated with this process is:

Na_2_SO_4_ + Ba(OH)_2_ 8H_2_O → BaSO_4_ ↓ + 2Na(OH) + 8H_2_O (3)

The mixture was maintained at room temperature and pressure under magnetic stirring for about 20 min, resulting in the precipitation of an off-white solid phase, and a transparent supernatant liquid. The solid phase was recovered by centrifugation for 5 min at 3000 rpm, equivalent to 1500g. The final product obtained was a lime putty with an approximate moisture of 50%, and the liquid phase was discarded. Afterwards, the precipitated solid phase, lime putty, was used as an element to manufacture the lime mortar.

#### Compositional Characterization of Lime

Lime used for the mortar manufacture was studied in fresh state as reported in [11,16]. The mineral composition of the samples was studied by X-ray diffraction (XRD, powder method) with Cu Kα radiation in a diffractometer (D8 Advance, Bruker, Karlsruhe, Germany) with a Bragg–Brentano θ geometry. Diffractometer settings were 40 kV, 30 mA, a scan range of 3–70° (2θ) with a step of 0.03° and a counting time of 1 s per step. Analysis of diffraction patterns was performed with the software X’Pert HighScore.

**Figure 1:**
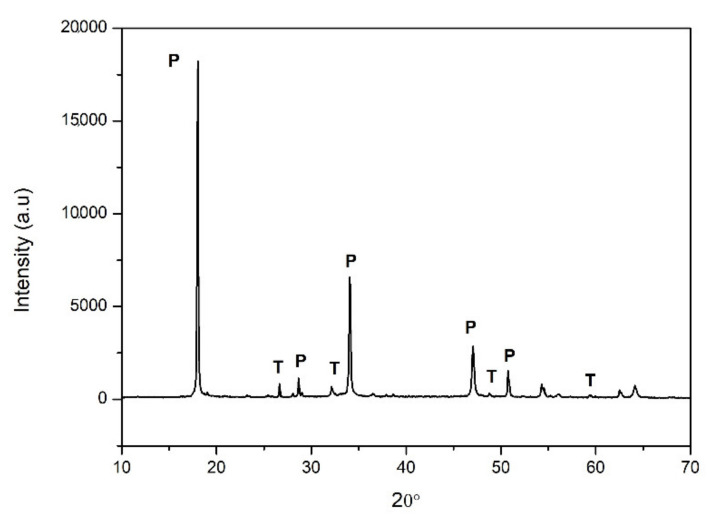
X-ray diffraction pattern of lime putty sample. P: Portlandite, T: Thenardite.

A diffractogram of a sample before soluble sulphates precipitation was registered for analysis by XRD (Figure 1). The XRD pattern reveals the major presence of portlandite and residual thenardite, as the result of incomplete phase separation. As below discussed, its transformation into barite is essential to avoid efflorescence in the mortars [16].

Another factor to be considered is the particle size distribution of this lime [16,17]. Particle size distribution was obtained by granulometry using water as dispersant and different sonication times: t = 0, 5, 10 y 15 min. As shown in Table 2, particle sizes are significantly larger than those obtained by field emission gun scanning electron microscopy (SEM-FEG) technique, which displayed an abundance of hexagonal portlandite crystals of tabular morphology (size ∼ 1 μm), very regular and well organized. This difference in sizes can be the consequence of an important lime agglomeration in the aqueous phase, with a decisive influence on the rheological behavior [11,12,13,14,15,16]. These differences are confirmed by the significant decrease in particle size in all the percentiles when the measurement was performed by applying ultrasound (see Table 2).

### 2.3. Mortars Manufacturing and Curing

M185 mortars were manufactured with lime putty obtained as described above and natural siliceous sand (98%) according to CEN Standard Sand EN 196-1 [18], with 1:3 lime/sand and 0.5 by weight water/lime ratios. These dosages coincide with the recommendations for lime masonry mortars indicated by ANCADE, Spanish association of lime and derivatives manufacturers [19]. They were prepared with a water content that allowed a consistency of 185 mm [20], measured in accordance with the UNE-EN 1015-3 standard [21] to ensure its workability [20]. M200 samples were prepared with a 0.54 by weight water/lime ratio, that allowed a consistency of 200 mm, in order to analyze the influence of water in the mortar properties and rheological behaviour.

Apparent density in the fresh paste was determined according to UNE-EN 1015-6/A1 standards [22] and air content according to UNE-EN 1015-7:1999 [23].

The prismatic specimens were prepared according to the UNE-EN 1015-2/A1 [24] and UNE-EN 1015-11 [25] standards. The samples were kneaded for no less than 3 min until they were completely homogenized and the set remained in the steel molds for 7 days under ambient conditions.

In addition, specimens were prepared to evaluate the behavior of the material with soluble sodium sulfate with expansive crystallization capacity (Group I) and with sulfates insolubilized by the addition of barium sulfate (Group II). (Table 3).

The properties of the fresh state are decisive, since they will greatly influence the final performance that the mortar will offer. The consistency test is considered as a measure of the fluidity and/or moisture of the fresh mortar. It provides information on the deformability or workability of the fresh mortar when it is subjected to a certain type of stress. The plastic consistency value is in the range of those of reference according to standard.

### 2.4. Control Tests and Analysis Techniques

#### 2.4.1. Thermogravimetric Analysis (TGA)

TGA profiles of fresh mortars were obtained in a Q-50 model (TA Instruments, New Castle, DE, USA), from room temperature to 600 °C, through a continuous increasing rate of 1 °C/min. The supposed water content in each sample was validated from TGA curves. Each thermal event was analyzed and different characteristic temperatures and parameters were estimated: T_onset_, the temperature at which degradation begins; T_final_, the temperature at which the event finishes; T_max_, the temperature at maximum derivative weight loss; ∆W, the weight loss, and the “residue” is the remaining undegraded sample after the test.

#### 2.4.2. Rheological Tests

Different rheological tests were carried out on fresh mortar samples to fully characterize both the viscoelastic properties and the viscous flow behavior of the mortars studied. Small-amplitude oscillatory shear (SAOS) tests were performed in a MARS controlled stress rheometer (Thermo-Scientific HAAKE, Darmstadt, Germany), using a 25 mm diameter roughened plate-plate geometry (3 mm gap). First, the extension of the linear viscoelastic regime (LVR) was determined by applying a stress sweep within the 10–1000 Pa range, whereas the mechanical spectra in the LVR were obtained through frequency sweep experiments within the range of 0.03–100 rad/s. On the other hand, viscous flow measurements were performed in a RS150 rheometer (Thermo-Scientific HAAKE, Darmstadt, Germany) by applying stepped shear rate ramps within the range 0.03–100 s^−1^, where shear rate was maintained in each step till steady-state values were achieved. In order to avoid slippage, phase separation, and fracture phenomena [26,27], a mixing geometry, dealing with a 37.15 mm diameter helical ribbon and a 46.62 mm diameter cup, was employed, calibrated according to the principles of the mixing rheometry technique [28]. Finally, creep measurements were performed in both rheometers by applying different stresses for 5 min to supplement the viscous flow curves at low shear conditions. The recovery behavior of the samples after the application of the stress was also evaluated for 5 min.

#### 2.4.3. Physical-Mechanical Test

To study the mortar mechanical properties, flexural and compressive strength tests were performed according to UNE-EN 1015-11 standard [25] with the help of a 300 kN multiservice press, from the Codein S.L. model MCO-30 (Control Desarrollo e Instrumentación, Madrid, Spain).

Flexural strength measurements were carried out on hardened prisms by three-point bending tests on standard geometry mortar specimens at the age of 28 and 90 days, in both mortar groups, In and IIn. Compressive strength tests were also performed on hardened mortar half-prisms after the bending tests, at the same ages (28 and 90 days). The same study was also carried out for Group Ia and IIa mortars.

For the characterization of the porosity of each mortar group, the real density was determined using a Helium pycnometer (model Pentapyc 5200e, Anton Paar, Graz, Austria); the open porosities were featured by means of mercury porosimetry (Pore Master 60 GT, Anton Paar), recording intrusion volume vs. pressure to obtain the pore diameter distributions from 900 µm to 0.0035 µm. The equipment used has two devices, one for low pressure (0.0–0.34 MPa) and other for high pressure (0.14–230 MPa) measurements. The low-pressure device is used to measure pore sizes greater than 7 µm. For smaller sizes, the high-pressure device is used, although the appropriate pressure range depends on the nature of the sample. The apparent density is determined by using the data obtained from the data recorded in PoreMaster control software as follows:

(4) $$\rho_{A}=\frac{m}{V_{\mathrm{pores}}+V_{\mathrm{holes}}+V_{\mathrm{real}}}=\frac{m}{V_{\mathrm{intruded}}+V_{\mathrm{real}}}$$

where m = sample mass, V_pores_ is the volume of pores, V_holes_ is the volume of holes, V_intruded_ is the total mercury intruded volume at the maximum pressure of the assay and V_real_ is the real volume of material.

Finally, to know the degree of compaction or disintegration of the material, the ultrasonic pulse velocity testing was applied. The ultrasound speed (V) was measured through the mortar by the direct transmission method using the UltraTest GmbH Bp-5 ultrasound kit, which is equipped with 50 kHz cylindrical transducers. The wave propagation was carried out in three directions: two parallel ones (V_x_, V_y_) and one perpendicular to the compaction plane of the prismatic specimens (V_z_). To calculate the total anisotropy (ΔM) and the relative anisotropy (Δm), Guydader and Denis’s formulae are used [29]:

(5) $$\Delta M=\frac{\frac{V_{x}+V_{y}}{2}\;\cdot {\;V}_{z}}{\frac{V_{x}+V_{y}}{2}}\cdot 100=\left(1-2\;\cdot \;\frac{V_{z}}{V_{y}+V_{x}}\right)\cdot 100$$

(6) $$\Delta m=\frac{V_{y}-V_{x}}{\frac{V_{x}+V_{y}}{2}}\cdot 100=\left(2\cdot \;\frac{V_{y}-V_{x}}{V_{y}+V_{x}}\right)\cdot 100\;$$

Measurements are made on specimens from Group In and Group IIn after different curing periods (20, 28, 32, 90 days). From a total of 15 measurements in each direction, the mean values were obtained and represented.

#### 2.4.4. Carbonation Monitoring

Portlandite is a highly alkaline phase while calcite is neutral; this change in alkalinity is used to detect the transformation from portlandite to calcite, consequence of the carbonation process of lime mortars. Thus, phenolphthalein tests were carried out to monitor the carbonation degrees of the different samples [30]. It is a quick qualitative technique, since it allows to know, in a certain and immediate way, if the sample contains only portlandite, in which case the sample acquires an intense purple-red color (pH > 9.5); if portlandite and calcite phases coexist, in which the sample acquires a soft pink hue (8 < pH < 9.5); or if only there is calcite, in which case a colorless area is observed (pH < 8). The depth reached by the pH zone below 8 (colorless zone) allows estimating the depth of carbonation. This technique is very useful to know where the carbonation front is and how it evolves throughout the curing time.

The transformation from portlandite to calcite has also been controlled during curing time by X-ray powder diffraction. For this purpose, two surfaces of fractured specimens, internal zone and external zone, have been analyzed. It is also possible to quantify the carbonation process (B) over time by the relationship between the height of the representative peak of calcite, I_a_ (θ = 29°), and portlandite, I_p_ (θ = 34°), as a function of the exposure time to natural carbonation [31]:

(7) $$B=\frac{I_{a}}{I_{p}}\;$$

This relationship gives an approximate quantitative assessment of the relative content of these phases in every sample. The entire study has been completed with textural observations by SEM with a 250 Zeiss EVO environmental scanning microscope (Carl Zeiss, Oberkochen, Germany), previously metallized with a thin layer of gold by sputtering.

## 3. Results and Discussion

### 3.1. Properties in Fresh State

#### 3.1.1. Consistency, Apparent Density and Entrained Air

The apparent densities of the mortars are directly related to those of their component materials, as well as to their air contents. They represent a relevant data that directly influences the viscosity and therefore the workability of the mixture. In our case, we observed densities of 2140 kg/m^3^ and 3% occluded air for M185 and 2120 kg/m^3^ and 3.25% occluded air for M200 [32].

#### 3.1.2. Thermal Analysis

TGA profiles confirmed the higher water content of the M200 sample (25.5%) in comparison with M185 (23.3%), as observed in Table 4. In addition, an outstanding thermal resistance of the mortars is inferred from these data since, apart from the occluded water, only around 4% weight loss was registered within the whole temperature range studied. This event, centred at 434 °C, is mainly attributed to portlandite decomposition. Further degradation of the mortars typically occurs at around 700 °C, where calcium carbonate starts to decompose [33].

#### 3.1.3. Rheological Characterization

**Figure 2:**
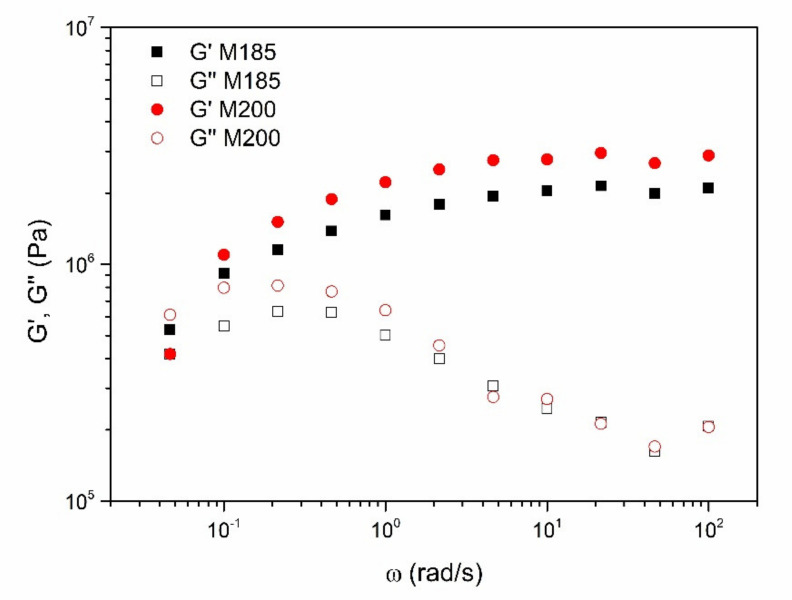
Evolution of the storage and loss viscoelastic moduli with frequency for both M185 and M200 mortar samples.

The linear viscoelastic range (LVR) of both mortars extends up to around 50–60 Pa (data not shown). The mechanical spectra obtained within the LVR are shown in Figure 2. In both mortars, the evolution of the storage (G’) and loss (G”) moduli with frequency is very similar, where a plateau region in G’ and a minimum in G” can be observed at high frequencies, characteristic of well-structured materials, whereas a tendency to reach the terminal region of the spectrum can be found at low frequencies. Moreover, G” values are almost coincident for both samples while G’ values are slightly higher in the case of M200. Even though this fact could be unexpected, the inclusion of a certain amount of water into the mortar can lead to a certain higher degree of rheological structuration. Although the addition of water usually leads to a decrease in mechanical strength of the mortars at the dry state [34,35,36], in the range of plastic consistency, it can lead to enhanced bond strength, thus increasing rheological properties in the fresh mixtures. Besides, these mechanical spectra were quite similar to those obtained previous to the sand addition, i.e., the phosphogypsum-based lime putty precursors, as previously reported [11].

**Figure 3:**
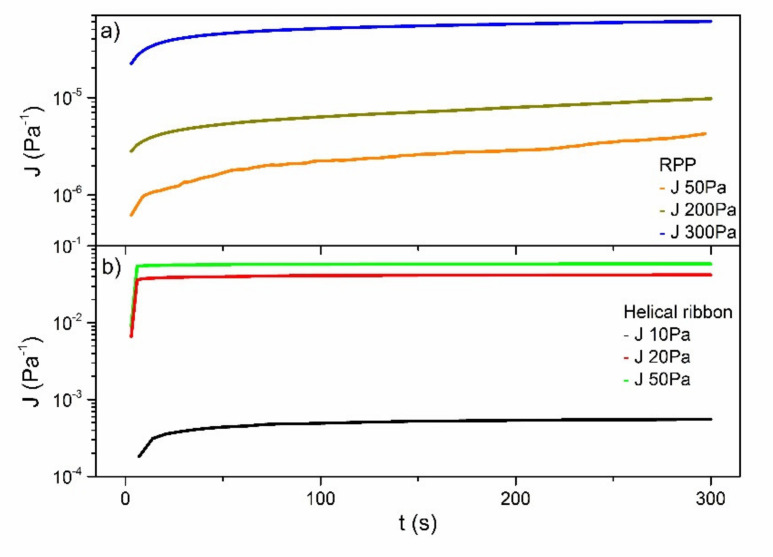
Compliance modulus evolution with time for M200 sample under different applied stresses using (**a**) rough plate-plate geometry and (**b**) helical ribbon geometry.

**Figure 4:**
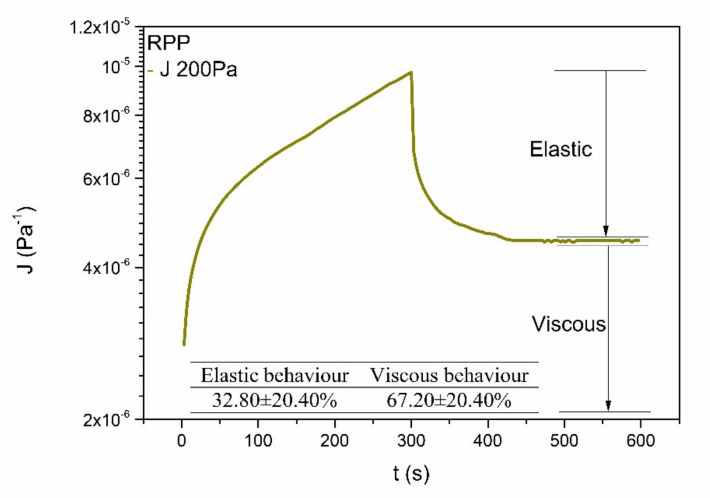
Selected creep and recovery tests showing the evolution of the compliance modulus with time when for M200 sample applying and releasing a constant stress. Inset: Average values of the elastic and viscous components of the sample considering all the different stresses applied.

Due to the very similar rheological response of both mortars, an additional viscous characterization was performed only in the sample M200. Creep tests were carried out by applying different constant stress values using both the traditional plate-plate geometry and the mixing geometry (Figure 3). In all cases, the creep compliance function, defined as the ratio of the transitory strain over the applied stress, increased with the stress, characteristic of the non-linear viscoelastic regime. However, significant differences were found in the stress values required to observe an appreciable flow using both geometries. While relatively high stress values (~50 Pa) are needed to achieve very small shear rate values, i.e., of the order of 10-7-10-6 s-1) using the plate-plate geometry, relatively low stresses (~10–20 Pa) produced much higher shear rates (in the range of around 10-5-10-4 s-1) when using the mixing geometry, which results in much higher values of the creep compliance. Besides, different maximum applicable stresses values are likewise obtained regarding both geometries (Table 5). These data reflect the importance of the measuring geometry in this type of samples, since it greatly influences the cohesiveness under non-static conditions. Despite this, comparable and consistent viscosity values were obtained with both geometries. Thus, from creep results, the apparent steady-state viscosity at very low shear rates can be estimated as the slope of the linear part of the curve:

(8) $$\eta =\frac{\Delta t}{\Delta J}$$

where *J* is the creep compliance function and *t* is time. Furthermore, the viscoelastic character of the mortar can be quantified through monitoring of the curve once the stress applied was removed. Thus, the two creep and recovery curves (as illustrated in Figure 4) allow to determine the relative viscous (or elastic) response. As can be seen, the mortar generally exhibits a predominant viscous response (67.2 ± 20.4%) but with a certain elastic component, for the range of stress values applied and the different measuring geometries employed.

**Figure 5:**
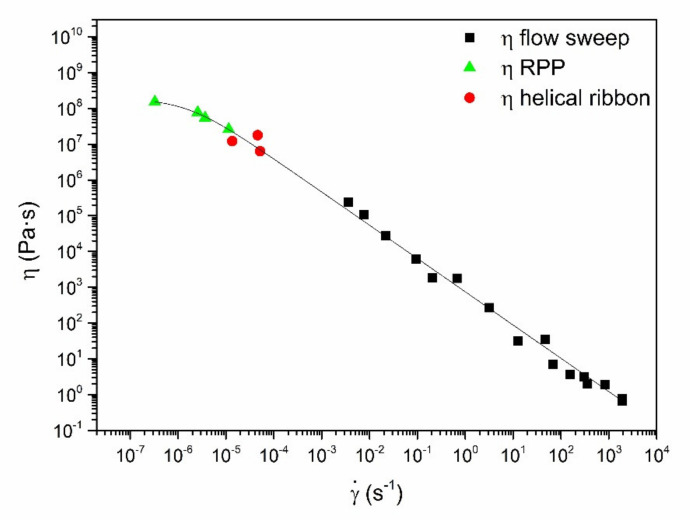
Viscous flow values for M200 sample including those obtained from compliance evaluation using both rough plate-plate (RPP) and helical ribbon geometries and those from viscous flow measurements (Cross model fit is also included).

By plotting the viscosity values obtained from creep experiments (Equation (8)) together with those measured in the stepped shear rate ramp tests, a complete viscous flow curve over 10 shear rate decades can be obtained. As shown in Figure 5, like a conventional lime mortar [37], a markedly shear thinning response, with a small tendency to achieve constant viscosity values at very low shear rates, is clearly apparent, which can be well fitted to the Cross model:

(9) $$\eta =\frac{\eta_{0}}{1+{\left(k\;\cdot \;\dot{\gamma }\right)}^{p}}\;$$

where $\eta_{0}$ is the zero-shear rate viscosity, k is a consistency parameter with time units, which gives an idea of the reciprocal shear rate at which the shear thinning character starts to take place, $\;\dot{\gamma }$ is the shear rate and p is the Cross rate constant, that establishes the degree of dependence of viscosity with the shear rate. In this sense, a p value of 0 indicates the system behave as a Newtonian fluid, while the stronger the shear thinning character, the closer the parameter to 1. Thus, the strong shear thinning character of the mortar is confirmed with the p value of 0.93, as well as with the very high zero-shear rate viscosity, as expected by the low values of the compliance modulus previously discussed (Table 5).

#### 3.1.4. The Microtextural Observations

The observation of the microstructure of the mortar samples in fresh state has been carried out by means of the analysis of images obtained using an environmental scanning electron microscope. It allows the observation of the mortar samples in fresh state, without any previous treatment, avoiding any change in the microstructure of the sample since once the mortar is dry, its properties are drastically altered [38].

**Figure 6:**
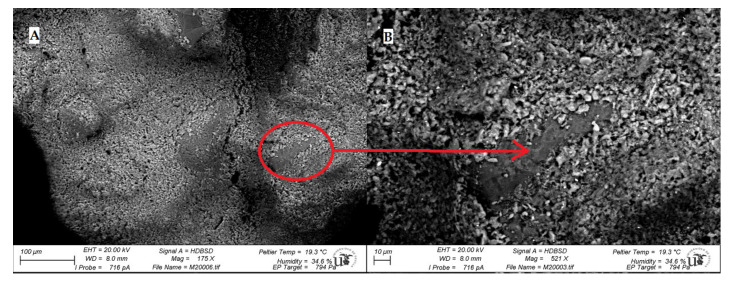
(**A**) SEM images of lime mortar paste (M200); (**B**) Detail of aggregate-putty adhesion.

Results of the M200 sample are shown in Figure 6A,B. It is observed that there is good internal cohesion within the paste. The aggregate-matrix interface (ITZ, Interface Transition Zone) of the lime mortar is the contact zone between the matrix and the aggregate grains that compose it. Figure 6B shows the matrix wrapping the aggregate. Adhesion between matrix and aggregate is mainly influenced by the texture of aggregate grains, the paste viscosity and the surface tension [39].

### 3.2. Hardened State Properties

#### 3.2.1. Influence of the Composition and Texture of the Mortar Specimens

Group I mortars are powdery to the touch, which indicates a poor cohesion in the carbonated matrix. When handled, they disintegrate and crumble very easily. This is the reason of their difficult demolding. However, Group I mortars acquire a higher degree of rigidity several days after demolding. A slight rounding of the edges, superficial wear, notable loss of material and development of superficial efflorescence due to migration of the salts towards the outside is also observed. This may account for the poor surface cohesion indicated above. On the contrary, Group II mortars offer a much more compact and more solid appearance. The specimens of this Group II acquire greater cohesion from the first days of their elaboration, which facilitates their removal from the mold.

**Figure 7:**
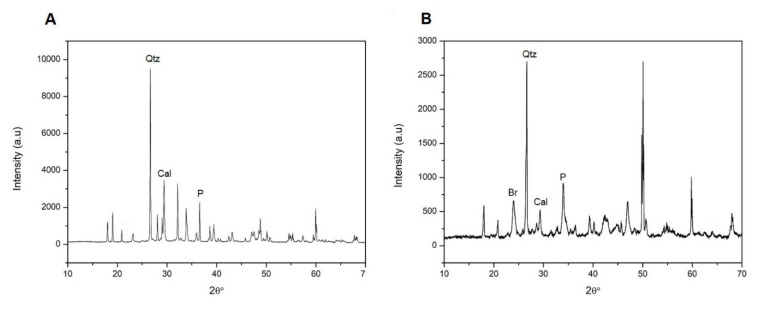
X-ray diffraction pattern of samples, after 90 days of curing. (**A**) Group In; (**B**) Group IIn, P: Portlandite, T: Thenardite, Cal: Calcite, Qtz: Quartz, Br: Barite.

On the other hand, in relation to the mineralogical composition of the mortars, it should be noted that, for all the samples analyzed, the mineralogy is very similar, as must be expected from the homogeneity of the mortar. Apart from the quartz as the majority phase of the aggregate (Figure 7), portlandite and calcite also appear, in variable proportions depending on the date of the tests, and thenardite for Group I mortars and barite for Group II mortars. The arrangement of crystalline neo-formation phases was not observed in any case.

**Figure 8:**
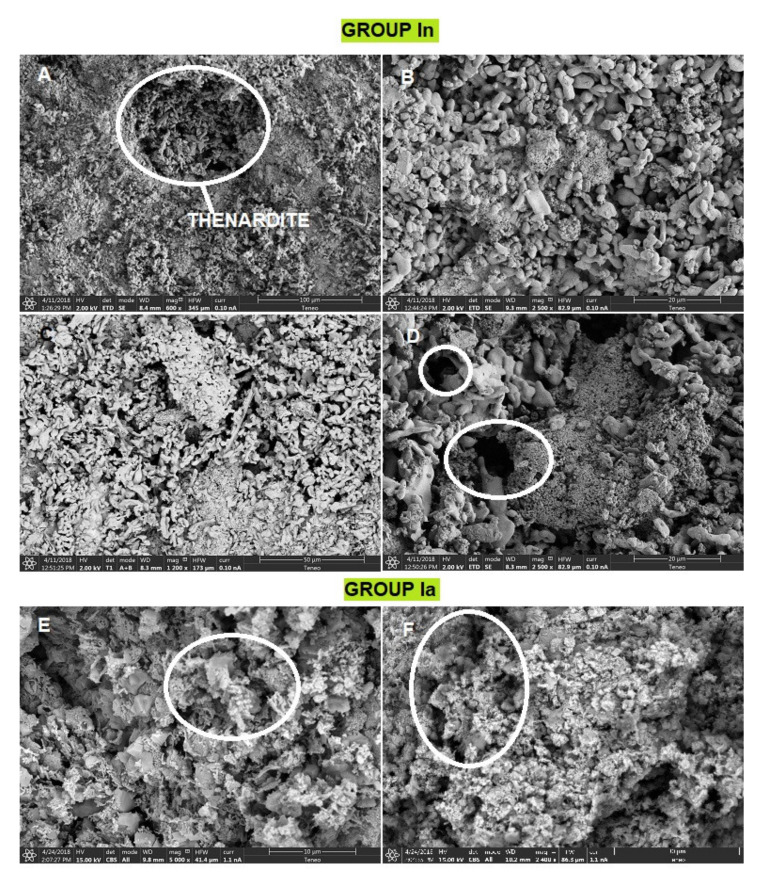
SEM images of group I mortar samples. (**A**) Thenardite crystallizations; (**B**) Detail of thenardite crystal morphology; (**C**) High porosity area; (**D**) Pores with a rounded morphology; (**E**) Carbonate matrix with calcite crystals (~2 µm); (**F**) Microcrystalline aggregates of calcite that seal pores and flaws.

Despite the homogeneity in their mineralogical compositions, the mortars showed clear textural differences. The SEM observations corroborated what has been described above. Group In mortars have a not very compact internal structure due to the dissolution and recrystallization of sulphates [40,41] and the high porosity is clearly observed (Figure 8A–D). The presence of big pores with a rounded morphology can be easily recognized (Figure 8A,D) and no signs of shrinkage are found. Figure 8E,F correspond to a mortar sample from Group Ia. A carbonate matrix is observed where two types of crystalline growth appear, calcite crystals (~2 µm) (Figure 8E) and microcrystalline aggregates of calcite that seal pores (Figure 8F) and flaws.

**Figure 9:**
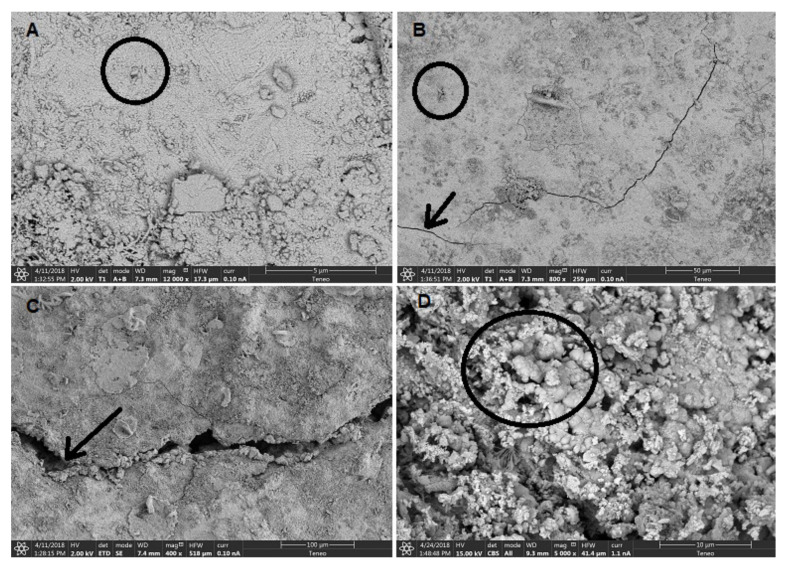
SEM images of group II mortar samples. (**A**) Compact internal structure with occluded pores; (**B**) Compact internal structure with occluded pores and fissures; (**C**) Small cracks detail; (**D**) Microcrystalline aggregates of calcite.

On the other hand, Group IIn mortars showed a much more compact internal structure, well interlocked and low porosity (Figure 9A–C). The presence of occluded pores is observed (Figure 9A,B) [42,43], possibly due to air bubbles trapped during kneading and setting. Small cracks also appear, possibly from hydraulic shrinkage or drying, which generates internal stresses that can cause volume reduction (Figure 9B,C) [44,45]. In Figure 9D, which corresponds to Group IIa, a carbonate matrix is observed, mainly formed by microcrystalline aggregates of calcite.

#### 3.2.2. Carbonation Mechanism of Mortar Specimens

**Figure 10:**
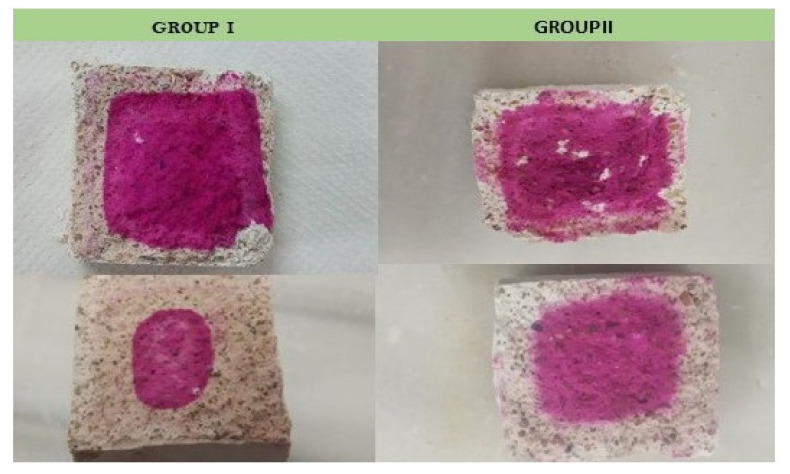
View of the fracture of representative specimens (40 × 40 × 40 mm^3^) of Group In and IIn cured for 28 days (upper), cured for 90 days (bottom).

**Figure 11:**
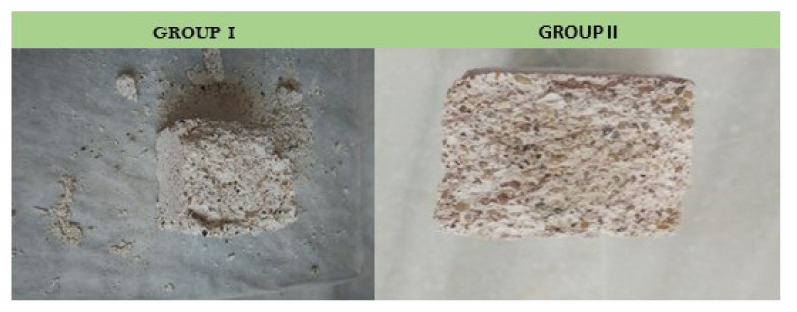
View of the fracture of representative specimens of Group Ia and IIa after 21 days in environmental chamber.

In general, an advance of the colorless zone towards the core of the specimen was observed over time for the specimens of both groups, although the way it was manifested is different in each case (Figure 10). Group In mortars are characterized by the formation of rings of different shades. The carbonation front is not uniform, which is apparent as an alternation of concentric bands. Both bands indicate areas rich in either portlandite or calcite. In those of Group IIn, the wave front is homogeneous, which is what is normally expected in this type of process, that the carbonation front progresses uniformly from the surface towards the interior [46,47]. The pattern of bands or rings observed in Group In mortar specimens is known as Liesegang rings. It is typical of periodic precipitation processes [48,49,50] and has been observed in the carbonation processes of lime mortars, especially those made with aged lime paste [51]. The reasons for the formation of the Liesegang pattern in the Group In specimens, and not in those of Group IIn, have yet to be determined with certainty, although they must be related to the respective porous structures and the presence of sodium or barium sulphate in Group In and Group IIn respectively. The morphology of those of Group In is more regular than that of Group IIn. In the former it is observed how the advance is parallel to the faces of the specimen, although with a smaller width on the face on which it rests. The advance of the front makes the area no longer perpendicular, being oval. In Group IIn mortars, the way the area is presented is quite irregular and it maintains its irregularity over time. On the other hand, in the accelerated carbonation series (Figure 11) the surfaces are colorless, which indicates that the specimens are completely carbonated and there is only calcite (colorless area).

The depth of carbonation with time is also studied using the solution of Fick’s diffusion law [52], in the form:

(10) $$x=h\cdot \sqrt{t}\;$$

where *x* is the depth expressed in mm, *t* is the time expressed in years and *h* is the advance speed of the carbonation front. Once *h* has been calculated as a function of time, depths at different ages of the mortars can be predicted. To estimate this depth, the phenolphthalein test applied to the cut faces of the mortar specimens is used. By means of this test, the carbonation depth is calculated by measuring it directly in the specimen, estimating the CO_2_ penetration depth as the average of the thicknesses measured around the sample [53].

The depth of carbonation data obtained at different times are collected in Table 6. In view of the results and considering that the number of data does not allow a rigorous statistical study, it is observed that the advance of the carbonation front presented a linear trend with t^½^ for the different types of mortars. The resulting *h* values are higher for Group In mortars (*h* = 26 ± 5 mm/year ^0.5^) and then for Group IIn (*h* = 18 ± 5 mm/year ^0.5^), due to the recrystallization process of sodium sulphate, which causes the deterioration and micro-cracking of the mortar, thus facilitating the access of CO_2_ to the interior of the structure. These data are in agreement with those obtained throughout the study.

**Figure 12:**
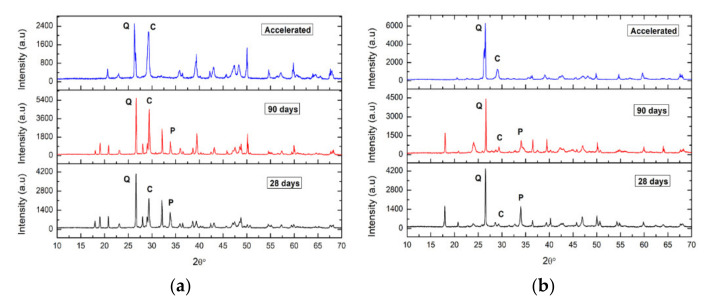
Graph that represents the evolution of carbonation over time in the area closest to the surface of a mortar from Group In (**a**). Graph that represents the evolution of carbonation over time in the area closest to the surface of a Group IIn mortar. (**b**). C: Calcite, P: Portlandite, Q: Quartz.

Samples are taken from both the internal and external areas of the specimens. The diffractograms of the areas closest to the surfaces of the fractured specimens after 28 days of curing show calcite peaks, for both the specimens of Group In (Figure 12a) and Group IIn (Figure 12b). In the fractured specimens after 90 days of curing, a decrease in portlandite peaks with a corresponding increase in calcite peaks was observed. Finally, the absence of portlandite in the accelerated carbonation specimens, Group Ia and Group IIa, indicates their total carbonation.

**Figure 13:**
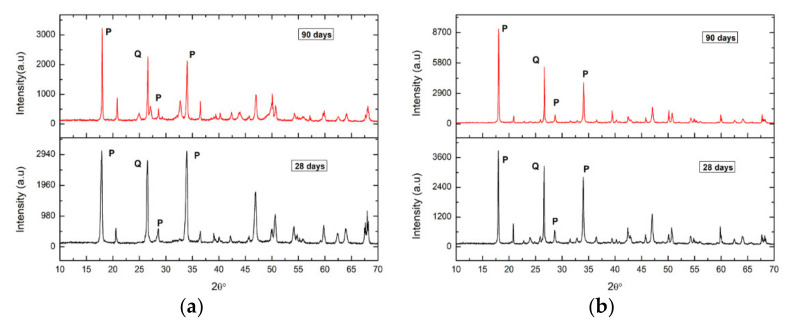
Graph that represents the evolution of carbonation over time of the internal zone of a mortar for Group In (**a**). Graph that represents the evolution of carbonation over time of the internal zone of a mortar for Group IIn (**b**). P: Portlandite, Q: Quartz, C: Calcite.

On the contrary, the diffractograms of the internal areas of the specimens (Figure 13) show, for both Group In and Group IIn, the absence of calcite in both the specimens cured at 28 days and those of older age (90 days). This relationship gives an approximate quantitative assessment of the relative content of these phases in the sample. The predictable content of CaCO_3_ to the detriment of Ca(OH)_2_ has been observed in all the samples over time [54], in the areas closest to the surfaces of the fractured specimens, since in the internal areas there has been no calcite detected. It is observed in Group In mortars that calcite is already the majority phase in the younger sample (28 days) in which more than 68% of portlandite has been transformed into calcite (Table 7). However, in Group IIn mortars, the majority phase is portlandite, although the calcite peaks that appear indicate a transformation of 23%. In both groups of specimens, the B values increase with the exposure time, as the expected evolution corresponds. Thus, the transformation of portlandite into calcite is faster for the Group In specimens than for those of Group IIn.

#### 3.2.3. Ultrasound Measurements

**Figure 14:**
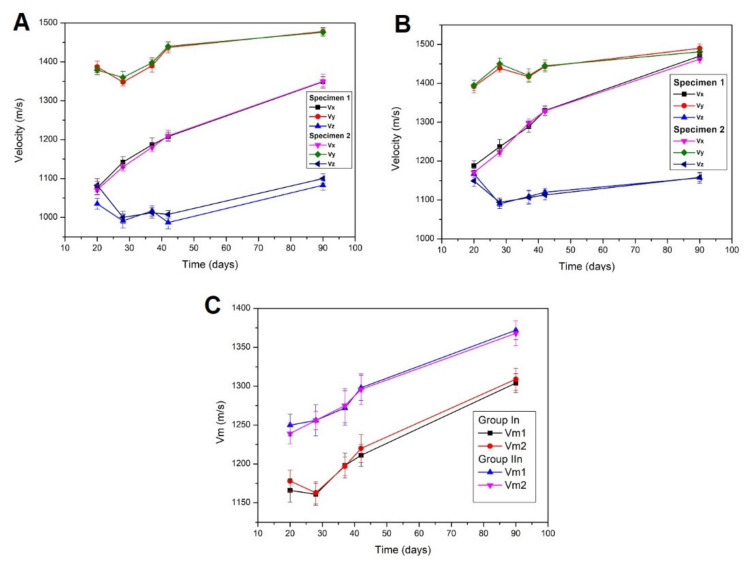
The ultrasonic speeds in the different types of mortars with natural carbonation Group In (**A**), Group IIn (**B**) where each value of V (Vx, Vy, Vz) corresponds to the mean value of the measurements taken in each direction of space. Mean Vm of the velocities is represented (**C**).

The lowest values, in both groups of specimens, are those of *V_z_*, which is the direction perpendicular to the compaction plane. This may be due to the formation of bubble planes or air pockets during manufacture along this direction. On the contrary, the highest values correspond to the directions *V_x_*, *V_y_* which are parallel to the compaction plane (Figure 14). 

In general, it is observed that the ultrasound speeds are low in the first days, in all cases, due to its poor internal structuring, which is also evident in its erratic behavior up to 37 days of study. After two months, the mortar hardens, producing an increase in speeds in the three directions of space, which may be attributable to the increase in internal structuring, i.e., calcite formation is taking place, without reaching its limit value because carbonation is still incomplete [20,45,55]. Regarding the mean values, *V_m_* (Figure 14C), it is observed that, in no case, do they exceed 1400 m/s at 90 days. This indicates the accomplishment of a homogeneous matrix due to the sealing of the pores and cracks due to the formation of calcite, since the molar volume of this is 37 cm^3^/mol compared to 33.1 cm^3^/mol of portlandite and 1 mol of CaCO_3_/mol Ca (OH) _2_ [56]. The highest velocity values are found in Group IIn mortars. The speed of sound in a medium material increases with increasing rigidity and/or specific volume. Thus, these data indicate a higher rigidity and, therefore, a more regular structure of mortars containing barium sulphate. Certainly, the medium is less porous, that is, denser, which contributes to a decrease in speed, so that, in this concurrence of effects, the increase in stiffness predominates. It can also be concluded that, since the ultrasound speed is lower in Group In mortars, they have a less compact and more porous structure than those of Group IIn [57].

Regarding the anisotropies, both total (ΔM) and relative (Δm) have not provided conclusive results (Table 8), i.e., a clear trend is not observed, due to the shortage of study times.

In general, for times longer than 28 days, the highest values correspond to the total anisotropy, for both Group In and Group IIn mortars, which indicates that there is a more marked anisotropy between the velocity perpendicular to the compaction plane and the parallel to this plane, which is interpreted as the consolidation of slight discontinuities along the *z* axis. Furthermore, it can be noted that the highest values of relative anisotropies occur in the earliest trials (Table 8). Therefore, the internal structure is more unstable and disorganization is greater, possibly due to the fact that the mortar is carbonating and the mixing water evaporating, causing it to shrink. Then the anisotropy is reduced as the mortar stabilizes, until it becomes constant when all the mixing water has evaporated, stopping the retraction, even with incomplete carbonation [42]. One aspect, which is generally not considered, is the contribution of the deterioration of the mortar by crystallization of the salts contained to the anisotropy. Thus, the specimens of Group In present greater total anisotropy than those of Group IIn. This crystallization, as indicated, generates flaking and cracks, which increase the anisotropy.

#### 3.2.4. Mechanical Properties

The flexural and compressive strengths of the mortars were also evaluated. The data of the corresponding tests are shown in Table 9. Regarding the flexural strength, for both the specimens of Group I and Group II, it increases with time. For those of Group In, an increase of 100% of the specimens with 90 days compared to those of 28 days, due to progressive carbonation, and 40% of those of the analogues of Group IIn is reported. These values are, on the other hand, clearly higher than their Group In counterpart. The accelerated carbonation period indicates the time in which all the samples were completely carbonated. The specimens of Group IIa present a maximum value of 2.6 N/mm^2^. This result is undoubtedly due to the presence of greater areas of structural weakness in the specimens of Group Ia, and therefore easier to break under exerted pressures.

Compressive strength also increases with aging, although always to a lesser extent in Group I mortars than in Group II. The maximum resistance to compression is obtained in Group IIa mortars, 4 N/mm^2^. These data agree with the ease of its removal, since they acquire consistency in the first days, and with the observations with SEM. Therefore, the importance of the effect of sulphate precipitation on material cohesion, reducing the deterioration of porous materials, is again evidenced [57].

## 4. Conclusions

(1) The negative influence of sulphates, present in PG, on the hardening of the lime putty obtained from PG treatment as well as on the mechanical properties of the resulting mortars has been demonstrated.
(2) Once precipitated the soluble sulphates, no anomalies on the lime putty were observed in the mortar carbonation processes, which proceeds in a similar way to conventional lime mortars.
(3) In spite of the sulphate contents of the lime putties before being treated with Ba(OH)_2_ are low enough to be undetectable by XRD, the difference in the performance of the mortars with and without previous Ba(OH)_2_ treatment highlights the importance of their elimination.
(4) The rheological behavior of the lime putty is like that described for conventional lime mortars. The parameters obtained for the two samples studied are similar, with little influence of water/lime ratio within the workability ranges. In fact, the higher water/lime ratio leads to a better rheological structuring of the fresh mortar, although this could result in a loss of mechanical properties in the hardened state. The creep response is highly influenced by the measuring geometry. A markedly shear thinning response, with a small tendency to achieve constant viscosity values at very low shear rates, is clearly apparent over 10 decades of shear rates.
(5) The formation of insoluble barium sulphate replacing soluble sodium sulphate increases mechanical strength and durability of the mortars.
(6) The mechanical strength of the specimens increases with the curing time due to the calcite formed that seals the pores as it is evidenced by the increase of the ultrasonic propagation speeds.
(7) The mortar manufacturing process gives rise to anisotropy of the speed of the ultrasound caused by the formation of stacking planes parallel to the compaction plane.
(8) The carbonation process progresses from the outside to the inside of the specimens through the porous network (pores, cracks and fissures).
(9) Liesegang patterns were observed in mortars containing rests of sodium sulphate. This phenomenon seems to be due to alternating calcite and portlandite precipitation. These patterns were not observed in mortars containing barium sulphate precipitate, indicating that the natural carbonation is controlled by diffusion-precipitation process.

## Figures and Tables

**Table 1:** Major chemical components and traces of the phosphogypsum sample. The X-ray fluorescence (XRF) data was normalized to the calcined mass. Uncertainties are given as standard deviations. Average activity concentrations in Bq/kg of the different radionuclides present.

Majority Elements	Composition (wt.%)	Concentration (Bq/kg)
SiO_2_	2.52 ± 0.03	^226^ Ra = 568 ± 25
Al_2_O_3_	0.20 ± 0.01	^210^ Pb = 722 ± 33
Fe_2_O_3_	nd	^40^ K < 111
MnO	nd	^232^ Th = 4 ± 1
MgO	nd	^235^ U = 19 ± 3
CaO	32.00 ± 1	
Na_2_O	0.01 ± 0.01	
K_2_O	0.02 ± 0.01	
TiO_2_	nd	
P_2_O_5_	0.65 ± 0.02	
SO_3_	46.00 ± 3	
Cl	nd	
F	nd	
SrO	nd	
BaO	nd	
LOI	18.40 ± 0.4	

LOI: Loss on ignition at 1000 °C, nd: not detected.

**Table 2:** Percentiles and particle sizes of the lime diluted in water for different ultrasound application times.

Ultrasound Application Time	Percentile
0 min	d(0.9) = 99.725 µm
	d(0.5) = 42.584 µm
	d(0.1) =14.926 µm
5 min	d(0.9) = 72.091 µm
	d(0.5) = 30.516 µm
	d(0.1) = 11.353 µm
10 min	d(0.9) = 45.614 µm
	d(0.5) = 19.686 µm
	d(0.1) = 4.856 µm
15 min	d(0.9) = 43.493 µm
	d(0.5) = 17.902 µm
	d(0.1) = 4.847 µm

**Table 3:** Sample groups and designations.

	Natural Carbonation (1)	Accelerated Carbonation (2)
Group I. Specimens Containing Na_2_SO_4_ Remaining from the PG	Group In	Group Ia
Group II. Specimens Containing Precipitated BaSO_4_	Group IIn	Group IIa

(1) 28 and 90 days in laboratory (25 °C of temperature and relative humidity 60%). (2) 21 days in an environmental chamber ((New Brunswick Galaxy 170), at 25 °C, 50–60% relative humidity and 10% vol CO_2_ concentration).

**Table 4:** TGA (Thermal Gravimetric Analysis) characteristics temperatures and main parameters of the thermal events displayed by both mortars studied (heating rate of 10 °C/min).

Sample	T_onset_ (°C)	T_max_ (°C)	T_final_ (°C)	∆w (%)	Residue (%)
M185	25/399	70/434	84/454	23.3/4.4	71.4
M200	25/405	62/434	76/449	25.5/4.4	69.2

∆w = weight variation.

**Table 5:** Maximum applicable stresses before sample rupture regarding both geometries used for M200 sample and fitting parameter values for the Cross model applied to the complete viscous flow curve.

Maximum Stress RPP	Maximum Stress Helical Ribbon	η_0_ (Pa·s)	k (s)	p
230–240 Pa	170–180 Pa	1.89 × 10^8^	638,811	0.93

**Table 6:** Depth values as a function of time for the different groups of samples.

Group	Time (Days)	Average Depth (mm)
Group In	28	5
Group In	90	13
Group IIn	28	3
Group IIn	90	9
Group In	(*h* = 26 ± 5 mm/year ^0.5^)	
Group IIn	(*h* = 18 ± 5 mm/year ^0.5^)	

**Table 7:** Ratio of intensities of the Ca(CO_3_) diffraction peak with respect to the Ca(OH)_2_ peak given by the parameter B for the In and IIn specimen groups of the zone closest to the surface.

GROUP IN		Group IIN	
B (28 days)	B (90 days)	B (28 days)	B (90 days)
2.1 ± 0.1 (68%)	4.2 ± 0.1 (81%)	0.3 ± 0.1 (23%)	0.6 ± 0.1 (38%)

**Table 8:** Total anisotropy (ΔM) and relative anisotropy (Δm) values of groups In and IIn mortar specimens for different time intervals elapsed since their demoulding.

	Group In (Specimen 1)		Group IIn (Specimen 2)	
Days	ΔM (%)	Δm (%)	ΔM (%)	Δm (%)
20	16 ± 2	25 ± 3	11 ± 2	25 ± 2
28	20 ± 2	16 ± 2	19 ± 2	18 ± 2
37	21 ± 2	15 ± 3	21 ± 2	16 ± 2
42	25 ± 2	17 ± 4	23.9 ± 1.5	17 ± 2
90	23.3 ± 1.5	9 ± 2	22 ± 2	8 ± 2
	**Group IIn (specimen 1)**		**Group IIn (specimen 2)**	
20	9 ± 2	16 ± 2	10 ± 2	17 ± 2
28	18 ± 2	15 ± 2	18 ± 2	16 ± 2
37	20 ± 2	9 ± 2	19 ± 2	9 ± 2
42	19.2 ± 1.4	8 ± 2	20 ± 2	8 ± 2
90	21.7 ± 1.5	1.3 ± 1.1	21.3 ± 1.3	1.3 ± 1.2

**Table 9:** Values of the flexion and compression tests for both groups of specimens Group I and Group II. The averages obtained for the specimens taken on the different test days for the different groups of specimens Group I and Group II are described.

**Natural Carbonation Process**				
	**Flexural Strength** **(N/mm^2^)** **28 Days 90 Days**		**Compressive Strength** **(N/mm^2^)** **28 Days 90 Days**	
In	0.3 ± 0.1	0.6 ± 0.1	0.8 ± 0.1	1.3 ± 0.1
IIn	1.0 ± 0.1	1.4 ± 0.1	1.2 ± 0.1	1.7 ± 0.2
**Accelerated Carbonation Process**				
	**Flexural Strength** **(N/mm^2^)** **21 Days on the Chamber**		**Compressive Strength** **(N/mm^2^)** **21 Days on the Chamber**	
Ia	0.8 ± 0.1		1.5 ± 0.2	
IIa	2.6 ± 0.1		4.0 ± 0.2	
